# Expert opinion on standard of care in pediatric malnutrition: a multidisciplinary perspective focusing on the context of ABCDs

**DOI:** 10.3389/fped.2026.1756490

**Published:** 2026-02-26

**Authors:** Omer Faruk Beser, Kursat Bora Carman, Fugen Cullu-Cokugras, Buket Dalgic, Aydan Kansu, Mehmet Kantar, Hasan Ozen, Hasan Tekgul, Bulent Unay

**Affiliations:** 1 Department of Pediatric Gastroenterology, Istanbul University-Cerrahpasa Cerrahpasa Faculty of Medicine, Istanbul, Türkiye; 2 Department of Pediatric Neurology, Eskisehir Osmangazi University Faculty of Medicine, Eskisehir, Türkiye; 3 Department of Pediatric Gastroenterology, Gazi University Faculty of Medicine, Ankara, Türkiye; 4 Department of Pediatric Gastroenterology, Ankara University School of Medicine, Ankara, Türkiye; 5 Department of Pediatric Oncology, Ege University Faculty of Medicine, Izmir, Türkiye; 6 Department of Pediatric Gastroenterology, Hacettepe University Faculty of Medicine, Ankara, Türkiye; 7 Department of Pediatric Neurology, Ege University Faculty of Medicine, Izmir, Türkiye; 8 Department of Pediatric Neurology, Gulhane Faculty of Medicine, Ankara, Türkiye

**Keywords:** anthropometry, deepening malnutrition, growth and development, growth charts, MCT, pediatric malnutrition, peptide-based formula, protein-rich formula

## Abstract

This review by a multidisciplinary panel of pediatric gastroenterology, pediatric neurology, and pediatric oncology experts aimed to address the standard of care in pediatric malnutrition in a context of ABCDs: A- Anthropometric assessment, B- Etiology-based evaluation, C- Nutritional Intervention & Treatment and D- Individualization & Restoration. The proposed standard of care in pediatric malnutrition involves routine assessment of growth and development at every pediatric visit, timely diagnosis and etiological classification of malnutrition, selection of optimal nutritional product meeting specific energy and protein requirements (such as energy- and protein-rich formulas with proteins constituting at least 10% of total calories), and implementation of appropriate nutritional intervention strategies tailored to the type and severity of malnutrition. These strategies may include stabilization, catch-up growth, nutritional rehabilitation, and restoration treatment using peptide-based enteral formulas, depending on the clinical context.

## 1 Introduction

Pediatric malnutrition (undernutrition) is a highly prevalent global issue with potentially irreversible effects on physical growth and cognitive development in children under the age of five. It is also associated with adverse health outcomes in middle and late childhood, as well as long-lasting consequences extending into adulthood (1–4).

American Society for Parenteral and Enteral Nutrition (ASPEN) defines pediatric malnutrition as an imbalance between nutritional requirements and intake, resulting in cumulative deficits of energy, protein, or micronutrients with likely adverse effects on growth, development, and other relevant outcomes (i.e., immunosuppression, infections, increased mortality risk) (5). The classification of malnutrition is based on etiology (primary, secondary), duration (acute, chronic), anthropometrics (stunting, wasting, and underweight), and severity (mild, moderate, and severe) (5).

According to World Health Organization (WHO) Indicators 2022, the global prevalence of stunting and wasting among children under five was reported as 22.3% and 3.6%, respectively (6). The Turkish Demographic and Health Survey (2018) indicated that 6% of children under the age of five are stunted, and 1.7% are wasted (7).

Primary malnutrition results from a multitude of factors, including poverty, inadequate maternal nutrition, low birth weight, suboptimal breastfeeding practices, inappropriate complementary feeding, food insecurity, recurrent infections, and environmental enteropathy (4, 8). In contrast, secondary malnutrition arises from the direct or indirect effects of underlying diseases on nutritional status and growth, such as prolonged severe infections, neurological disorders, malignancies, congenital heart disease (CHD), chronic kidney disease (CKD), chronic liver disease (CLD), malabsorption syndromes, immune deficiencies, and cystic fibrosis (4, 8, 9).

Effective management of pediatric malnutrition involves regular nutritional screening, accurate identification of malnutrition type and severity, assessment of nutritional support needs, and timely, individualized intervention (4, 10–12). When implemented correctly and by disease-specific indications considering factors such as route, formula composition, dosage, duration, and monitoring — nutritional support can significantly improve clinical outcomes, reversing malnutrition and mitigating its adverse effects (4, 5, 10–12). Nevertheless, malnutrition often remains underdiagnosed or underestimated in pediatric clinical settings, contributing to higher morbidity and mortality, delayed recovery, prolonged hospitalization, and increased healthcare costs (4, 5, 11–13). This is particularly relevant for neurologically impaired children, given that monitoring nutritional comorbidities and implementing appropriate nutritional support have become an integral part of their care with rising prevalence of this group of children presenting feeding difficulties and gastrointestinal symptoms, often leading to malnutrition (14–17). They are also the most common group requiring enteral feeding alongside the long-term management issues due to complexity of comorbid gastrointestinal problems related to oral motor function and motility and nutritional complications (9, 12, 14–17).

Despite all measures, childhood malnutrition remains one of the most significant problems in the world, and given the current political climate, it is not expected to decrease in the coming years. Besides, there is a need for standardized protocols with a clearly defined set of core outcomes to be integrated into research and clinical practice workflows to optimize guideline-concordant patient care in the management of childhood malnutrition (1).

In this review, we have attempted to summarize the management of malnourished children based on the current literature, prioritizing children with neurological diseases who most frequently require nutritional support in childhood, and to propose a model of care intended for use in our country as a basis for updating the current state of clinical practice. For this purpose, an algorithm for standard of care in in pediatric malnutrition management was proposed by a panel of experts in pediatric gastroenterology, neurology, and oncology, addressing the anthropometric assessment, etiology-based evaluation, nutritional intervention and treatment and the individualization and restoration with special emphasis on energy and protein requirements, the bidirectional relationship between malnutrition and gastrointestinal dysfunction, and therapeutic strategies for chronic and progressive malnutrition.

## 2 Methods

A multidisciplinary panel of experts in pediatric gastroenterology, neurology, and oncology convened to form a consensus on the standard of care in pediatric malnutrition. The panel searched PubMed/Medline, Scopus, and Web of Science from inception to September 2025 for potentially relevant articles including international guidelines, consensus statements, systematic reviews, meta-analyses, randomized controlled trials (RCTs), population studies, and multicenter cross-sectional studies that have focused on diagnostic anthropometry, etiological assessment, and optimal nutritional interventions in pediatric malnutrition. As supported by scientific evidence and expert clinical opinion, the proposed framework outlines the essential pillars of standard of care in pediatric malnutrition management, based on the following Algorithm 1 in a context of ABCDs (A- Anthropometric assessment, B- Etiology-based evaluation, C- Nutritional Intervention & Treatment and D- Individualization & Restoration):

Algorithm 1 **Standard of Care in Pediatric Malnutrition Management**

A. Anthropometric Assessment in Pediatric Population

 • Perform routine growth and development monitoring:

  - Track weight, height, mid-upper arm circumference (MUAC), triceps skinfold thickness (TSFT), and z-scores against standardized growth charts.

  - Conduct nutrition-focused physical examination.

  - Include psychometric evaluation when relevant.

B. Etiology-Based Evaluation of Pediatric Malnutrition

 • Identify the underlying cause(s) of malnutrition:

  - Inadequate intake (e.g., feeding difficulties, poor appetite)

  - Increased losses (e.g., malabsorption, chronic diarrhea)

  - Increased demand (e.g., systemic illness, inflammation)

C. Nutritional Intervention & Treatment

 • Initiate nutritional therapy based on clinical indications:

  - Follow the four-phase recovery model:

   1. Stabilization

   2. Recovery

   3. Active catch-up

   4. Nutritional rehabilitation

 • Monitor clinical and nutritional response throughout.

D. Individualization & Restoration

 • Tailor nutritional intervention to individual clinical needs:

  - Use energy- and protein-rich formulas to meet specific needs.

  - Consider peptide-based formulas to improve tolerance, absorption, and gut restoration when polymeric formulas are not tolerated.

 • Adjust intervention dynamically based on progress and reassessment.

## 3 Anthropometric assessment in pediatric malnutrition

A comprehensive nutritional assessment (i.e., nutrition-related medical history, medication history, physical examination, anthropometry and laboratory data) is essential for early detection of malnutrition and timely commencement of individualized nutritional interventions (13, 18, 19).

### 3.1 Trending anthropometric data in relation to growth charts and z scores

Growth is considered the gold standard tool and primary outcome measure of nutritional status in pediatrics (11, 13). Growth should be monitored at regular intervals throughout childhood and adolescence and measured every time a child presents, regardless of the health setting (i.e., preventive, acute, or chronic care) (5, 13). In this regard, anthropometric measurement is the initial critical component of all primary care routine well visits in pediatric clinical practice (18, 20). Accurately collecting and trending anthropometric data over time and reviewing age and gender-adjusted growth charts at every pediatric visit is essential in early recognition of failure to thrive (FTT), particularly a decline in weight percentile, and malnutrition (5, 12, 18).

Anthropometric measures of growth include weight for age (WFA), length for age (LFA), weight for length (WFL) in children under 2 years of age and head circumference up to 36 months of age, while standing height for age (HFA), WFA and body mass index (BMI) for age are typically collected after 2 years of age (12, 13, 21).

Based on anthropometric measurements, malnutrition can be classified as severe, moderate and mild (4, 8, 12) (Table 1).

**Table 1:** Multimodal approachment for diagnosis and categorization of pediatric malnutrition (12, 13, 26, 27).

WHO growth charts	Mild malnutrition	Moderate malnutrition	Severe malnutrition
Weight for age (Gómez classification) *acute malnutrition*	75 – < 90% of the norm	60 – < 75% of the norm	< 60% of the norm
Length/height for age (Waterlow classification) *chronic malnutrition*	90 – < 95% of the norm	85 – < 90% of the norm	< 85% of the norm
Weight for length/height (up to 36 months) *acute malnutrition*	-	< 10th percentile	< 5th percentile
BMI for age (2- 20 years)	< 5th percentile (*undernutrition)*		
Head circumference	Decrease in rate of growth *(developmental delay with malnutrition)*		
Z scores	Mild malnutrition	Moderate malnutrition	Severe malnutrition
Weight for length/height	−1 to −1.9	−2 to −2.9	−3 or greater
BMI for age			
MUAC			
Multiple data points	Mild malnutrition	Moderate malnutrition	Severe malnutrition
Weight gain velocity (≤2 years of age)	<75% of the norm	<50% of the norm	<25% of the norm
Weight loss (2 to 20 years of age)	5% usual body weight	7.5% usual body weight	10% usual body weight
Deceleration in weight for length/height or BMI for age z scores	Decline of 1 z-score	Decline of 2 z-score	Decline of 3 z-score
Inadequate nutrient intake (% of estimated energy/protein need) (≤2 years of age)	51–75%	26–50%	≤ 25%

BMI, body mass index; MUAC, mid-upper arm circumference.

Weight velocity (for children under 2 years; useful for early detection of abnormal growth patterns), head circumference (0–36 months; developmental delays may be associated with malnutrition), MUAC (in children aged ≥2 months), and TSFT (in children >12 months) are additional anthropometric parameters used to assess growth and development (11, 12, 22) (Table 1).

Although changes in growth percentiles are visually easy to interpret on a growth chart, current guidelines recommend using z-scores instead of percentiles for anthropometric assessments. Z-scores provide more precise information on a child's nutritional status by quantifying how far individual measurements deviate from population medians (5, 11–13, 23). A decline in z-scores—reflecting a greater negative deviation from the median—is associated with an increased risk of severe malnutrition (5).

When compared to WFL, WFH and BMI z-scores, MUAC z-scores are considered to be more sensitive to diagnose mild-to-moderate malnutrition and to track the change in nutritional status overtime, and also to provide more reliable data in the presence of ascites or edema, being not affected by fluid shifts or hydration status (24–26). Recently, MUAC z-score tape was developed to further facilitate the diagnosis of primary malnutrition which enables single-step assessment of nutritional status (as defined by z-score) without using ancillary reference charts and calculators, and for a larger number of children regardless of the any age and across a wide weight range (12, 26–28).

Currently accepted z-score ranges differentiate mild malnutrition (z-score −1 to −1.9), moderate malnutrition (z-score −2 to −2.9), and severe malnutrition (z-score ≤ −3), which can be further characterized as acute changes (<3 months) or chronic (>3 months) in duration (5, 29) (Table 1).

According to ASPEN recommendations, the diagnosis of pediatric malnutrition requires evidence of deterioration in at least one of the following indicators, assessed using data from two separate time points: weight gain velocity (for children aged <2 years), weight loss (for those aged >2 years), deceleration in weight-for-length or BMI-for-age z-scores, or inadequate nutrient intake (13, 30, 31) (Table 1). These criteria are applicable when multiple anthropometric data points are available over time, and a decline in any single indicator may be sufficient to establish the diagnosis of malnutrition.

### 3.2 Anthropometric assessment of malnutrition in children with neurological disability

In children with neurological disabilities, such as those with cerebral palsy (CP), the accuracy of anthropometric measurements is often compromised by factors like joint contractures, muscle atrophy, and movement disorders. These limitations reduce the reliability of standard anthropometric tools and may lead to misinterpretation of nutritional status (12, 32–34).

Furthermore, the commonly used cut-off values below −2 z-scores on standard growth charts for the healthy children—typically applied for nutritional assessment and malnutrition classification—may not be appropriate for children and adolescents with CP. This is due to differences in growth trajectories between children with CP and typically developing peers, which necessitates individualized interpretation and, in some cases, alternative growth references (13, 19, 35, 36).

Accordingly, nutritional assessment of children with neurological impairment is suggested to include the evaluation of body composition (assessment of body fat mass and muscle wasting) besides the weight and height measurements (37–39). Nutrition-focused physical exam (NFPE), including the evaluation of muscle and subcutaneous fat wasting, signs of oedema, oral health, suck, swallow/breathe ability, appetite and affect, is considered useful in providing supportive evidence to malnutrition in these children (38–41).

Developmental assessment and neurocognitive monitoring are recognized as key functional outcome measures in the context of malnutrition. In contrast, restricted growth and stunting are considered incomplete proxies for developmental delay, and should be interpreted with caution (5, 11, 31, 42).

## 4 Etiology-based evaluation of pediatric malnutrition

The diagnosis of malnutrition necessitates further evaluation to identify the underlying etiology, which is classified into three main categories: inadequate nutrient intake, increased caloric losses or malabsorption, and increased systemic caloric demand. These causes may be related to an underlying organic medical condition, non-illness causes, or a combination of both (18, 43, 44).

### 4.1 Inadequate nutrient intake

In most cases, inadequate nutrient intake is related to non-illness causes such as lack of parental knowledge of appropriate feeding, patient feeding refusal or other maladaptive feeding behaviors, food insecurity, and less commonly parental abuse or neglect (18, 45). The remaining cases of inadequate nutrient intake are attributed to underlying organic medical conditions such as acute illness, gastrointestinal, endocrine, genetic and other systemic inflammatory disorders (18, 44) (Table 2) (Table 2).

**Table 2:** Etiology-based evaluation of malnutrition (14, 39–42).

Etiology of malnutrition		
Inadequate nutrient intake		
Primary malnutrition	Nonorganic/non-ilness causes	Lack of parental knowledge of appropriate feeding practice Patient feeding refusal, other maladaptive feeding behaviors Poverty and food insecurity Parental abuse or neglect
Secondary malnutrition	Organic causes (underlying medical condition)	Acute illness Gastrointestinal, endocrine, genetic, and other systemic inflammatory and non-inflammatory disorders
	Risk factors for an underlying medical condition and hospitalization need	History of prematurity More severe decline in weight/height z-score (z-score of <−3)
Increased caloric losses/malabsorption		
Secondary malnutrition	Organic causes (underlying medical condition)	Gastrointestinal disorders causing malabsorption: Inflammatory bowel disease, celiac disease, food-protein allergy, exocrine pancreatic insufficiency such as cystic fibrosis, and other protein-losing enteropathies
Increased systemic caloric demand		
Secondary malnutrition	Organic causes (underlying medical condition)	Chronic diseases such as congenital heart disease, kidney or liver disease, anemia, malignancy, and chronic infectious diseases

The risk factors increasing the likelihood of underlying medical condition and hospitalization for evaluation and initial treatment in a malnourished child are history of prematurity and a more severe decline in weight/height z-score (z-score of <−3) (18, 46) (Table 2).

### 4.2 Increased caloric losses/malabsorption

The underlying conditions in this category typically are gastrointestinal conditions that result in malabsorption, such as inflammatory bowel disease, celiac disease, food-protein allergy, exocrine pancreatic insufficiency such as cystic fibrosis, and other protein-losing enteropathies. These conditions can also all be exacerbated by nonorganic factors (18) (Table 2).

### 4.3 Increased systemic caloric demand

In this category, it is challenging to consume enough calories to meet the increased demand due to underlying chronic diseases such as CHD, CLD, CKD, anemia, malignancy, and chronic infectious diseases. Nonorganic factors can also exacerbate these conditions (18) (Table 2).

## 5 Nutritional intervention & treatment

The critical components of pediatric malnutrition care include timely recognition of children at risk of malnutrition, the accurate identification of malnourished children, appropriate selection of nutritional products in terms of delivery method and required intake, and the implementation of admission and discharge criteria, treatment duration, and monitoring protocols (4).

### 5.1 Indications for initiating nutritional support and indicators of eventual recovery

Nutritional support is the cornerstone of malnutrition treatment, with two primary goals: restoration of cellular function (short-term) and replenishment of lost tissue (long-term) (4, 47–49). The criteria for initiation and monitoring of nutritional support are summarized in Table 3 (1, 4, 49–52).

**Table 3:** Nutritional support onset and recovery criteria in pediatric malnutrition (1, 4, 45–48).

Indications for onset of nutritional support
• Failure to receive <60% to 80% of the nutritional requirements for >10 days • Total feeding time for >4–6 h per day • Insufficient oral intake for more than 5 days (>1 year of age) or more than 3 days (<1 year of age) • Wasting and stunting: - Lack of weight gain or improved height during monthly follow up for children < 2 years of age - Failure to gain weight or presence of weight loss during follow-up visits in 3 months for children > 2 years of age - Decline of more than 2 major percentile lines on growth charts - Triceps skinfold consistently below the fifth percentile of age - Decreased height velocity of ≥0.3 z-scores per year, or a reduction greater than 2 cm per year during puberty
Nutritional support is continued until recovery criteria met
• Weight-for-height reaches 90% of the expected value or • Weight-for-height z-score reaches −1

In children achieving a WFH ≥ −1 z score or ≥ 90% of the median WHO reference values under nutritional support, the progress is considered (4, 52). However, most children remain in the range of −1 to −2 z scores within the specified time limits (max 6 months) of the first nutritional support and they eventually regress to −3 z scores without continued nutritional support. Moreover, even in those with progress, there is a high risk of relapse of malnutrition (1, 4, 52).

Hence, the participating experts consider that current clinical nutrition practice only covers the children with established malnutrition, while those with mild malnutrition (−1 to −2 z scores) remain at risk of worsening to established malnutrition particularly in the setting of primary malnutrition, and those with chronic or severe malnutrition remain insufficiently treated given their need for a prolonged nutritional support. In general, adherence to nutritional product is higher in secondary malnutrition, while discontinuation rates are remarkably high in children with primary malnutrition who were prescribed with home nutritional support.

Accordingly, periodic monitoring of the child every 3–6 months during the first 2 years following recovery or discharge is essential. An effective strategy should also be implemented to track children who miss follow-up appointments, as they are at increased risk of malnutrition recurrence (1, 4, 52–54). At each visit, caregivers should be asked about the child's recent health status, feeding practices, and play activities, and the child should be thoroughly assessed for growth and developmental progress (1, 4, 52).

### 5.2 Stabilization, active catch-up and nutritional rehabilitation

Malnourished children with a good appetite (documented to be taking at least 80% of the recommended daily oral supplement), and those without clinically noticeable medical complications are eligible for outpatient management with oral supplement support (55).

Conversely, those who have a poor appetite (consuming below the minimum standard) or present with complications should be referred and admitted for inpatient care or stabilization centers (55).

Children with severe acute malnutrition (SAM) and complications require hospitalization. The nutritional support principles are similar in primary and secondary malnutrition, which involves stabilization, active catch-up and nutritional rehabilitation. The inpatient care is discontinued when WFH/L z score or MUAC z score ≥ −2 and edema has completely resolved for at least 2 weeks (4, 55). Failure to regain appetite and weight or reduce edema are the criteria for declaring “failure to respond to treatment,” which requires extensive medical evaluation and return to the stabilization phase (4, 8, 55) (Table 4).

**Table 4:** Stabilization, active catch-up and nutritional rehabilitation phases of severe acute malnutrition (4, 5, 81).

Stabilization phase
A cautious approach is required with
• initiation of feeding as soon as possible • decreasing the feeding frequency gradually • use of nasogastric tube feeding - in anorexic children - in those with oral intake <80 kcal/kg/day (aged <5 year) or <80% of recommended energy intake
Catch up growth
Starts when the energy intake is >150 kcal/kg/day with use of ready to use therapeutic food (RUTF) or WHO recommended formula in young children (6–59 months)
Rehabilitation
Starts with return of good appetite and reduction of edema – leads to discontinuation of treatment with recovery of anthropometric indices used for admission
Discharge criteria in hospitalized patients
• Weight for length/height or MUAC z score reaches ≥–2 • No edema for at least 2 weeks
Failure to respond treatment
Considered in case of failure to regain appetite or reduce edema – mandates extensive medical evaluation and return to the stabilization phase

Appropriate nutritional intervention is a critical component in the management of malnutrition (9, 18, 56). Experts emphasize the following primary goals in the treatment of pediatric malnutrition:

Timely replenishment of protein, energy, and micronutrient deficits through individualized nutritional support tailored to each patient's specific needs, including the duration and method of intervention
Promotion of catch-up growth
Support of neurodevelopmental and immune system maturation
Incorporation of a “restoration phase” alongside replenishment in the treatment plans for children with chronic, progressive malnutrition

### 5.3 Energy requirements

Recommended energy intake for the healthy children and in those with specific underlying conditions is summarized in Table 5 (4, 22, 57–59).

**Table 5:** Recommended energy (4, 18, 53–55).

Healthy children		Recommended energy intake (kcal/kg/day)
Age		
0–3 months		102–110
4–6 months		82–84
6–12 months		78–82
13–35 months		81–83
3–8 years	Boys	60–85
	Girls	60–85
9–18 years	Boys	36–47
	Girls	34–40

Mild-to-moderate malnutrition is treated generally on an outpatient basis with increasing the amount of energy intake by 50%–100% of that recommended for the age-matched healthy children (57–59).

In infants, breastfeeding is continued along with enriched supplementary feeding and addition of enteral product when necessary (4, 57).

Daily energy requirement for catch-up growth in children with primary malnutrition is calculated based on the condition of the malnourished child with 1.2–2.0-fold higher energy intake than recommended for the healthy children (4, 58, 59) (Table 5).

In children with secondary malnutrition, energy requirement is determined based on the underlying disease with consideration of higher energy intake for hypermetabolic conditions (i.e., chronic disease, severe infection) and lower energy need in those with minimal activity (i.e., children with neurological disease, bed-ridden children) (4, 8) (Table 5).

### 5.4 Protein requirements

In the general pediatric population, sufficient protein intake is necessary in infancy and early childhood periods to enable normal growth and development (22, 60).

As shown in Table 6, recommendations for protein intake in the pediatric population as well as the terminology related to nutrition vary depending on the issuing health authority. Dietary reference values (DRVs) and Dietary Reference Intakes (DRIs) are umbrella terms for a set of nutrient reference values. Estimated Average Requirement-EAR/Average Requirement-AR refers to the level of nutrient intake that is adequate for half of the people in a population group. Recommended Dietary Allowance-RDA/Population Reference Intake-PRI refers to the level of nutrient intake that is adequate for virtually all (97%–98%) people in a population group. Adequate Intake-AI refers to the average observed daily level of intake by a population group/groups of apparently healthy people that is assumed to be adequate. Tolerable Upper Intake Level-UL refers to the maximum amount of a nutrient that can be consumed safely over a long period of time), and Lower Threshold Intake-LTI refers to the level of intake below which almost all individuals will be unable to maintain “metabolic integrity”, according to the criterion chosen for each nutrient (60–66).

**Table 6:** Current recommendations for protein requirements, estimated by age and sex, for children (56–59, 62).

Age		European Food Safety Authority (EFSA)			Dietary Reference Intakes (DRI)			
		AR (g/kg bw/d)	PRI (g/kg bw/d)	PRI (g/d)	EAR (g/kg bw/d)	RDA (g/kg bw/d)	RDA (g/d)	AMDR (%E)
7–12 months					1.0		11.0+	
1–3 years					0.87		13.0	10–30%
4–8 years		0.72	0.89	19.30	0.76	0.95	19	10%–30%
9–13 years		0.72	0.90	34.50	0.76	0.95	34	10%–30%
14–17 years	Boys	0.71	0.88	53.25	0.73	0.85	52	10%–30%
	Girls	0.69	0.85	46.50	0.71	0.85	46	10%–30%

AMDR, Acceptable Macronutrient Distribution Range; AR, Average Requirement; EAR, Estimated Average Requirements; PRI, Population Reference Intake; RDA, Recommended Dietary Allowance; bw, body weight.

### 5.5 Energy and protein-rich formula

The Acceptable Macronutrient Distribution Range (AMDR) for protein is considered to be 5%–20% of total calories for children during 1–3 years of age and 10%–30% of total calories for children 4–18 years of age (67). As age increases, the protein content of the formula per 100 kcal generally increases, and lower calorie formulas have a higher percentage from protein. According to their caloric density, formulas are classified as low-/isocaloric (<0.9 kcal/mL), normocaloric/ (0.9–1.2 kcal/mL), and high energy (>1.2 kcal/mL) formulae (68, 69).

Accordingly, experts consider that the standard of care in pediatric malnutrition involves use of an energy and protein-rich formula, which is defined as the formula containing ≥1.2 kcal/mL and ≥ 4 gr protein/100 mL. Protein should constitute at least 10% of total calories (70, 71).

Nonetheless, it should be noted that beyond the dietary recommendations to prevent deficiency, there are no guidelines for an “optimal” protein intake in pediatric population for promoting healthy growth and development (60). In fact, protein intake trends in children and adolescents in Western Europe and United States are usually two- to three-fold higher than the dietary recommendations (60). The transition period to a family diet is considered a critical time window for protein intake, as usually characterized by a rapid increase of protein intake mainly due to the shift to cow's milk which has two times higher protein content (5.15 g/100 kcal) compared to infant formula (IF) or follow-on formulas (FOFs) (60, 72–74). This seems notable given that increased intakes exceeding the current recommended protein intake in the pediatric age group are considered likely to be causally related to an increased risk of obesity across one's lifespan (60, 73–75).

Hence, the European Food Safety Authority (EFSA) scientific opinion on the composition of IFs and FOFs recommended that the minimum level of protein in these formulas should remain at 1.8 g/100 kcal, but that the upper limit for protein content of FOFs should be reduced from 3.0 to 2.5 g/100 kcal (71, 76, 77).

However, these recommendations are based on the factorial method utilizing nitrogen balance studies, which tend to overestimate nitrogen intake and underestimate excretion, leading to a net positive balance and potentially underestimating the actual protein requirements of children and adolescents (60, 62, 78). Therefore, further research is needed to clarify critical time windows, define optimal protein intake ranges, and validate current protein intake guidelines in the pediatric population (60).

### 5.6 Lipid content: MCT, as an additional energy source containing formula

Although an increase in the energy density of foods and thus provision of adequate energy in diet is often achieved by increasing the lipid content, there is disturbed lipid metabolism in children with severe malnutrition (10, 79). While healthy children need approximately 30% of their daily energy intake from fat after the age of 2, the recommended amount is higher for malnourished children. The WHO recommends F75 with 32% of its total energy coming from fat at the beginning of malnutrition treatment while recommends F100, that has 53% of its total energy coming from fat, for the rehabilitation period (52). MCTs are easier to digest, absorb, and metabolize than LCTs, which makes them a preferable source of abundant and rapidly available energy in case of increased energy needs (i.e., undernourished patients after major surgery or children during normal or retarded growth) (10, 80–83). However, to avoid essential fatty acid deficiency, care should be taken to ensure that energy from long-chain fatty acids does not fall below 15%.

## 6 Individualized/personalized treatment

Individualized/personalized treatment aims to provide optimal treatment meeting specific energy and protein requirements with respect to etiology-based evaluation (underlying chronic disease) and severity of malnutrition.

### 6.1 Increasing nutritional needs of children with chronic diseases

The recommended protein requirements for the pediatric critical care population (ASPEN Clinical Guidelines), children with normal growth, metabolism, body composition, and activity levels (National Academy of Sciences), and underweight infants and children requiring catch-up growth (National Research Council) are summarized in Table 7 (13, 63, 75, 84).

**Table 7:** Protein requirements (recommended dietary allowances) (13, 71, 80, 81).

Children with normal growth, metabolism, body composition, and activity	
0 to 6 months	1.52 g/kg/day
6 to 12 months	1.2 g/kg/day
12 to 36 months	1.05 g/kg/day
4 to 13 years	0.95 g/kg/day
14 to 18 years	0.85 g/kg/day
>18 years	0.8 g/kg/day
Pediatric critical care population	
0 to 2 years	2 to 3 g/kg/day
2 to 13 years	1.5 to 2 g/kg/day
13 to 18 years	1.5 g/kg/day
Infants and children who are underweight and need to achieve catch up growth	
0 to 6 months	2.2 g/kg/day
6 to 12 months	1.6 g/kg/day
1 to 3 years	1.2 g/kg/day
4 to 6 years	1.1 g/kg/day
7 to 14 years	1 g/kg/day
15 to 18 y (males)	0.9 g/kg/day
15 to 18 y (females)	0.8 g/kg/day

Enteral nutrition (EN) remains the preferred route of nutrient delivery in critically ill children and, whenever feasible, should be initiated within the first 24–48 h of pediatric intensive care unit (PICU) admission. Current guidelines (85) recommend that parenteral nutrition (PN) not be initiated during the initial 24 h of critical illness. In children with an adequate baseline nutritional status and considered to be at low risk of nutritional deterioration, initiation of supplemental PN should be deferred until at least one week after PICU admission. Conversely, in malnourished or high-risk children, PN may be considered within the first week of admission if sufficient advancement of EN cannot be achieved (Table 8). However, in recent years, data have emerged suggesting that early initiation of PN should not be considered, regardless of the child's nutritional status (86, 87). Protein provision should be initiated early in the course of critical illness, with a minimum target of 1.5 g/kg/day, acknowledging that higher intakes may be necessary in infants and younger children, as well as in those with more severe illness, to promote a positive protein and nitrogen balance. This should then be gradually increased as tolerated up toward 3 g/kg by the stage of recovery with appropriate provision of energy. The safety of protein intake >3 g/kg/d in children has not been demonstrated (85, 88). Similar to the recommendation to start PN late, there are also data suggesting that it would be better not to give protein in the acute phase of parenteral nutrition (87).

**Table 8:** Nutrition support in pediatric critical care.

Nutrition support	Recommendation	Evidence/Strength
Initiation of EN	EN should be initiated in all critically ill children within 24–48 h, if not contraindicated.	Expert opinion, weak
PN – within first 24 h	PN should not be initiated within the first 24 h of PICU admission.	Moderate quality, strong
PN in well-nourished, low-risk patients	If EN is not feasible, initiation of PN should be delayed for at least 1 week after PICU admission.	Single RCT, weak
PN in malnourished/high-risk patients	In patients with malnutrition or at high risk of nutrition deterioration, if EN cannot be advanced beyond minimal volumes, PN may be considered within the first week.	Expert consensus, weak
Timing of protein delivery	Protein should be provided early in the course of critical illness to promote positive nitrogen balance.	Moderate quality, weak
Protein target	A minimum intake of ≥1.5 g/kg/day is recommended. Higher intakes may be required in infants and young children to achieve positive protein balance.	Moderate quality, strong

PN, parenteral nutrition; EN, enteral nutrition.

For protein and energy requirements for “optimal” catch-up growth; in the first step, it is necessary to decide what the ratio of fat and lean mass of the weight gain will be. Total body percent fat changes with age. If we want the desired catch-up growth to be 30% fat (normal total percent body fat of a 6-month-old infant) and 70% lean body mass (25% of which is protein), the additional energy requirement for 1 g of weight gain will be (0.3 × 9) + (0.7 × 0.25 × 4) = 3.4 kcal. If we aim for a 10 g/kg/day weight gain per day, we must provide 10 × 3.4 = 34 kcal/kg/day energy in addition to normal energy requirement (84, 89).

Similar calculations can be made for protein requirements. 20%–25% of lean body mass is protein. If we assume that the metabolic efficiency of dietary proteins is 70%, 0.36 g (0.25/0.7) protein should be taken for 1 g of lean body. If a 10 g/kg/day weight gain is planned, 3.6 g/kg protein should be given in addition to the maintenance protein requirement (84, 89).

Accordingly, as shown in Table 9, increasing nutritional needs (specific calorie and protein requirements) should be considered in certain disease groups associated with increased likelihood of malnutrition and growth delay, such as neurological disability, CKD, oncologic disease, CHD and cystic fibrosis (4, 5, 85, 88, 90–102).

**Table 9:** Increasing nutritional needs of children with chronic diseases.

Children with neurological disability (4, 31, 84, 86, 87)
• Protein requirement for catch-up growth, in addition to maintenance requirement, is 2 g/kg/day necessitating use of protein-rich formula • Isocaloric (<0.9 kcal/mL) formula to prevent overload or weight gain complicating the patient care • High calorie (>1.2 kcal/mL) formula only if volume restriction is needed • Fiber-containing nutritional products is recommended
Children with chronic kidney disease (4, 88–90)
• Energy requirement is 100% of the estimated energy requirement (EER) for chronological age • Dietary protein intake at 100% to 140% of the dietary reference intake (DRI) for ideal body weight
Pediatric oncology patients (4, 8, 91–93)
• Pediatric patients hospitalized with cancer suffer from protein and energy deficiency, and they receive insufficient daily oral energy and protein, which is only 63%–79% of the recommended daily intake • Nutritional support in pediatric oncology patients is recommended to be based on an energy-rich (120% of healthy children) and protein-rich formula with fiber • Tube feeding should be preferred for children with insufficient oral intake under chemotherapy whereas parenteral nutrition is recommended for those experiencing gastrointestinal complications or having less than 50% of daily intake via gastrointestinal tract • The increase in energy and protein requirement under chemotherapy should be taken into consideration with frequent low volume protein-rich and high-fiber feeding and energy intake at 120% of that recommended for the healthy children
Children with congenital heart disease (4, 94, 95)
• Energy intake should be 50% higher than that recommended for the healthy children for catch-up growth along with 2–4 g/kg protein intake. These children should consume 55%–60% of their caloric intake from carbohydrates and 30%–35% from fat
Children with cystic fibrosis (4, 96–98)
• Recommended daily calorie intake (RDA) is 120%–140% of the healthy children with proteins constituting 15% of total energy • Increased protein intake protects fat-free body mass and improve growth and long-term clinical outcomes

### 6.2 Restoration-focused nutritional intervention

#### 6.2.1 Bidirectional interplay between malnutrition and compromised gastrointestinal function

**Figure 1:**
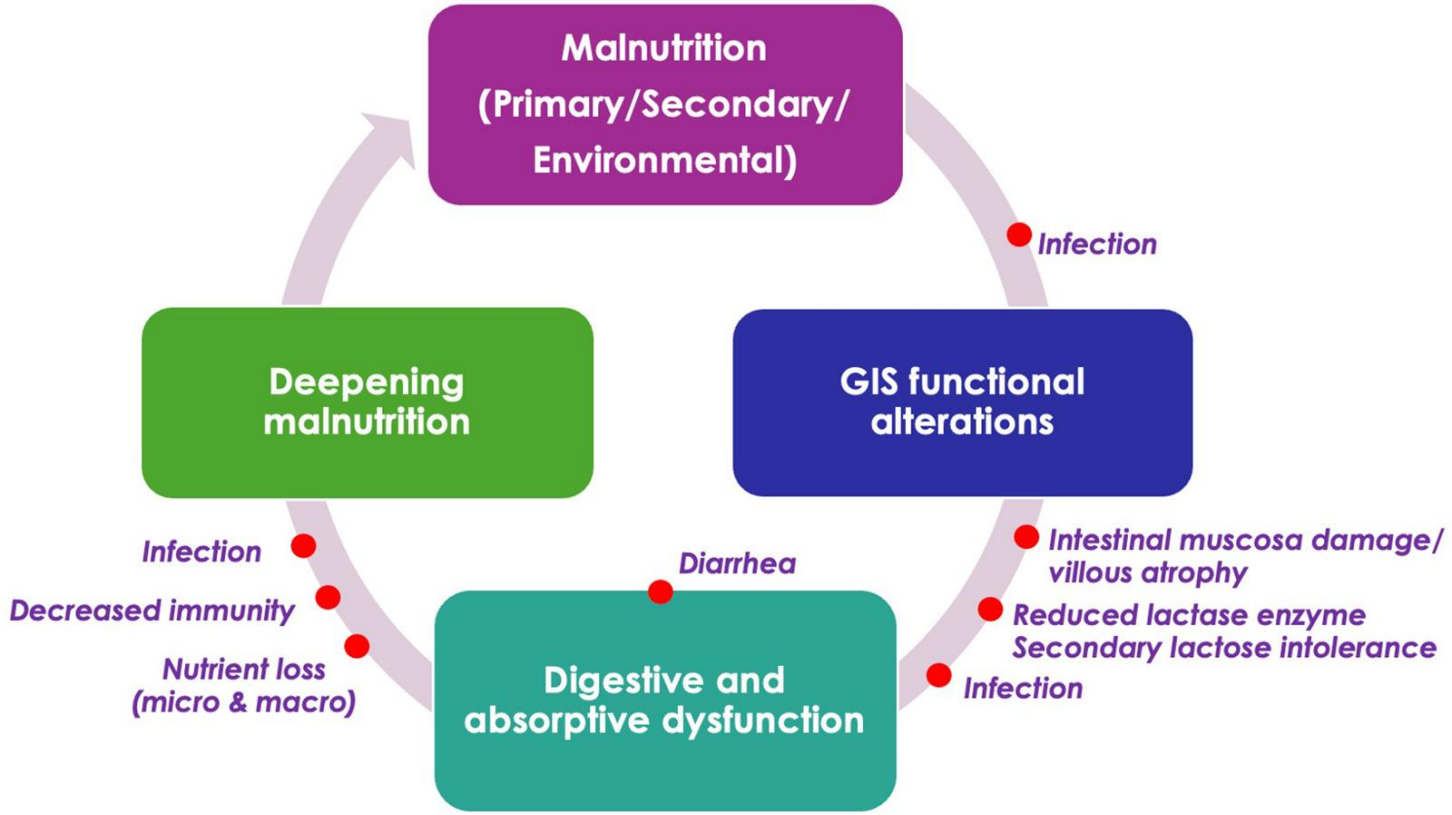
Interplay between malnutrition and gastrointestinal functional alterations. Adapted from Selimoglu et al. (10).

Malnutrition leads to alterations in gastrointestinal digestive and absorptive functions—such as reduced pancreatic exocrine activity, villous atrophy, increased intestinal permeability, loss of digestive enzymes, malabsorption, and diarrhea—which collectively reduce the effectiveness of nutritional support and may further exacerbate malnutrition (10, 103–105). Additionally, functional gastrointestinal alterations contribute to malnutrition by impairing gut function through structural and functional intestinal changes, immune activation, and growth failure. This bidirectional relationship highlights that gastrointestinal dysfunction can be both a consequence and a cause of malnutrition (10, 106) (Figure 1).

#### 6.2.2 Restoration: An optimal nutritional treatment strategy for deepening chronic malnutrition

Presence of gastrointestinal functional abnormalities is considered an important factor in selection of the best nutritional support, since compromised gastrointestinal function interferes with efficacy of nutritional support and recovery from malnutrition (10, 103, 107). Hence, appropriate nutritional care in malnourished children should address the overlapping and interacting effects of diarrhea, enteropathy and malnutrition to improve child survival and developmental potential in the long-term (9, 103, 108, 109). In this regard, in patients with deepening malnutrition, “restoration” becomes a critical nutritional target to enable normalization of gastrointestinal physiological functions and thus a maintained/restored gut integrity (10).

During this period, children should have improved appetites and mental state, no vomiting or diarrhea, be metabolically stable, gaining weight, and any edema should have reduced or completely disappeared. Children whose risk of refeeding syndrome has been eliminated can consume up to 220 kcal/kg of energy per day, and those who are breastfed should continue to breastfeed. Children should also receive social and emotional support (52).

#### 6.2.3 Using peptide-based enteral formulas for restoration in deepening malnutrition

Presence of impaired gut function with severe mucosal abnormalities and malabsorption in malnourished children limits the use of polymeric enteral formulas, which leads to deepening malnutrition in these patients due to decreased tolerability (10, 110). Dilution of formulas to achieve adequate tolerance and to prevent diarrhea is not an appropriate approach in malnourished children given that they are already nutritionally compromised, and this dilution results in lower levels of nitrogen intake and prolonged negative nitrogen balance (10, 110, 111).

Peptide-based enteral formulas may be considered within individualized nutritional plans to support improved tolerance, absorption, and restoration of gastrointestinal function, particularly when polymeric formulas are poorly tolerated or insufficient. By promoting recovery, they offer the potential to accelerate restoration and thus save valuable clinical time.

In malnourished children, deficiency of specific essential amino acids due to protein maldigestion and malabsorption can further worsen gastrointestinal mucosal atrophy and thus reduce protein absorption even further (112). However, peptide transport (absorption) is less severely affected by malnutrition than the free amino acid transport, possibly due to efficient and rapid uptake of di- and tripeptides compared with free amino acids (112). Accordingly, presence of specific peptide carrier systems in the intestinal brush border that are independent of free amino acid carrier systems is of critical importance in the effective treatment of malnourished patients with intestinal malabsorption, emphasizing the likelihood of improved gastrointestinal tolerance with peptide-based formulas (112).

The inclusion of protein in the form of small peptides (dipeptides and tripeptides) in peptide-based formulas appears to be advantageous, as these peptides are absorbed more rapidly and efficiently than free amino acids, and their absorption is less adversely affected in pathological states (10, 110, 111). In this context, peptide-based enteral formulas may offer clinical benefits in the nutritional management of malnourished children with compromised gastrointestinal function. Compared to free amino acid or whole-protein formulas, peptide-based formulas are associated with improved gastrointestinal tolerance and absorption, better nitrogen retention and balance, reduced incidence of diarrhea and bacterial translocation, enhanced fat absorption, and the maintenance or restoration of gut integrity (10, 110, 111, 113).

Participating experts agree that peptide-based enteral products can be safely utilized to address gastrointestinal dysfunction without imposing additional stress on an already compromised and fragile gastrointestinal system in the context of progressive malnutrition. This makes them a favorable nutritional option during the restoration phase of malnutrition treatment, prior to transitioning to polymeric enteral feeding. Accordingly, the use of an isocaloric, isoosmolar peptide-based formula enriched with medium-chain triglycerides (MCTs)—which provide rapid energy support—is considered essential for facilitating restoration through an optimized nutritional approach that improves nutritional status while preserving gastrointestinal integrity.

The experts claim that in children with severe malnutrition or gastrointestinal intolerance as well as in children with neurological impairment and cancer, isocaloric peptide-based formula enriched with MCT should be recommended as a standard of care in treating deepening malnutrition.

Indeed, besides compromised gastrointestinal function, transitional feeding is also considered amongst the indications for use of a peptide-based formula (10, 111, 113). Evidence about enteropathy in primary malnutrition is limited. However, a patient's clinical condition is crucial in determining the need for MCT-enriched peptide-based formulas, especially in cases of intolerance to polymeric enteral feeding or abdominal distension.

## 7 Standard of care in pediatric malnutrition: key components

The experts reached consensus on certain claims to improve pediatric malnutrition care, referred to as “standard of care” in pediatric malnutrition, which include:

Assessment of growth and development via trending anthropometric data plotted on the age- and gender-matched growth charts at every pediatric visit, in any health setting to recognize and intervene malnutrition, which is particularly critical within the first 5 years of life providing a golden window of opportunity for growth.
Timely repletion of protein, energy, lipid and micronutrient losses using the best appropriate method with optimal duration to enable catch-up growth and support of mental, motor and immune development, through an individualized nutritional support tailored to specific needs and type and severity of malnutrition.
Provision of optimal nutritional intervention meeting energy and protein requirements, using an energy and protein-rich formula containing MCT which is defined as the formula containing ≥1.2 kcal/mL and ≥ 4 gr protein/100 mL (constituting at least 10% of total calories).
Careful implementation of nutritional intervention principles (stabilization, active catch-up and nutritional rehabilitation) in patients with malnutrition, regardless of primary or secondary malnutrition.
Considering the bidirectional interplay between malnutrition and compromised gastrointestinal function in selection of the nutritional support that addresses the overlapping and interacting effects of diarrhea, enteropathy and malnutrition, since compromised gastrointestinal function at baseline interferes with efficacy of nutritional support and recovery from malnutrition.
Considering restoration, besides the repletion of loss, as a critical nutritional target in children with deepening or chronic malnutrition to enable normalization of gastrointestinal physiological functions and thus a maintained/restored gut integrity. Use of an isocaloric isoosmolar peptide-based formula enriched with increased MCT for this purpose as a standard of care in treating deepening malnutrition, to enable restoration of gastrointestinal functions without further stressing the fragile gastrointestinal system, before returning to standard enteral feeding.
Considering periodic monitoring of the child every 3–6 months during the first 2 years after progress (recovery) or discharge and tracing children who fail to attend follow-up appointments and thus are at increased risk of recurrence of malnutrition.
Considering the gaps in current clinical nutrition practice regarding that it only covers children with established malnutrition, while those with initial malnutrition (−1 to −2 z scores) remain at risk of worsening to established malnutrition, particularly in the setting of primary malnutrition, and those with chronic or severe malnutrition remain insufficiently treated given the prolonged nutritional support requirement.
Potential barriers to implementing the proposed standard of care in real-world clinical settings are likely such as resource limitations particularly in regions experiencing conflict, food insecurity and economic instability, as well as training gaps, and cultural considerations. Policy-driven strategies (i.e., optimizing resources allocations, promoting integrated care models, increasing training) should be developed and conducted by fully considering the social, cultural and community contexts to ensure the implementation of the proposed algorithm.
Preventing and appropriately treating malnutrition will prevent the development of many problems not only in childhood but also in adulthood. Although it has been a problem in our world for many years, there are still many unanswered questions; what is the most appropriate nutritional protocol and macro/micronutrient composition in malnutrition treatment, what will be the effect of treatments targeting the gut microbiota, what can/should be done to prevent malnutrition in children worldwide and how can the negative consequences of malnutrition in adulthood be prevented?.

## 8 Limitations

Childhood malnutrition remains a significant problem worldwide. However, the current literature is still lacking in terms of determining the most appropriate nutritional approach for its treatment. There is an insufficient body of research on the influence of intestinal microbiota in malnutrition treatment, the appropriate amounts and benefits of micro and macronutrients, and the ideal protein structure and fatty acid composition. Due to the limited space, the management of diseases other than neurological disorders that can cause malnutrition has not been discussed in detail.

## 9 Conclusion

In conclusion, this multidisciplinary review by experts in pediatric gastroenterology-hepatology-nutrition, pediatric neurology, and pediatric oncology outlines key components of a standard of care for pediatric malnutrition in a context of ABCDs, with a particular focus on diagnostic parameters to be integrated into routine pediatric visits and on providing optimal nutritional therapy tailored to specific energy and protein requirements. The proposed standard of care emphasizes:

**A. Anthropometric assessment:** Routine anthropometric assessment with respect to growth and developmental milestones at every pediatric encounter; considering special circumstances in neurologically disabled children (preferring evaluation of body composition and using NFPE given the reduced reliability of standard anthropometric tools and lack of alternative growth references).

**B. Etiology-based evaluation:** Timely diagnosis and etiological classification of malnutrition; considering the monitoring nutritional comorbidities as the critical component of care in neurologically disabled children.

**C. Nutritional intervention & treatment:** Selection of appropriate nutritional products that meet individual energy and protein needs—preferably using formulas in which proteins constitute at least 10% of total caloric intake; and adherence to established nutritional intervention principles, including the phases of stabilization, catch-up growth.

**D. Individual/personalized treatment with etiology-based approach:** In the context of etiology-based personalized care, peptide-based formulas enriched with MCT may be preferred for patients with progressive malnutrition or poor tolerance to polymeric formulas, to support gastrointestinal restoration. This is particularly relevant for neurologically impaired children, as the most common group requiring enteral feeding, given the complexity of comorbid gastrointestinal problems and nutritional complications leading to long-term management issues.
